# Poland syndrome: Fortuitous discovery of a familial case in Togo

**DOI:** 10.1016/j.ijscr.2023.108281

**Published:** 2023-05-03

**Authors:** Tamassi Bertrand Essobiyou, Kossi Albert Labou, Tete Edem Kouevi-Koko, Pegwende Rachid Cedric Diendere, Ekoue David Dosseh

**Affiliations:** a General Surgery Department, Sylvanus Olympio University Hospital Center, Lome, Togo; b Neurosurgery Department, Sylvanus Olympio University Hospital Center, Lome, Togo; c Radiology Department, Charles de Gaulle Hospital, Ouagadougou, Burkina Faso

**Keywords:** Poland, Agenesis, Pectoral, Thorax, Togo

## Abstract

**Introduction and importance:**

Poland syndrome is a rare malformative disease. It is characterized by agenesis or hypoplasia of the pectoralis major muscle associated or not with a malformation of the ipsilateral thoracic limb. The authors report the fortuitous discovery of a familial case of Poland syndrome in Togo.

**Case presentation:**

He was a 25-year-old young man, with no known pathological history, examined as part of a physical fitness assessment and who presented with thoracic asymmetry. The clinical and radiological explorations made it possible to conclude to a Poland syndrome in its minor form without any other associated malformation. In addition, three other paternal uncles of the patient presented with the same clinical symptomatology. In the absence of a clear indication, the patient received no treatment.

**Clinical discussion:**

Poland syndrome is a rare congenital malformation. The abnormalities encountered are cutaneous-glandular, osteo-cartilaginous and muscular. The etiopathogenesis of the disease is unknown. The diagnosis is clinical and is based on the demonstration of agenesis of the pectoralis major muscle. The disease does not often lead to functional discomfort; therefore the treatment is not systematic and has only aesthetic value.

**Conclusion:**

A rare congenital disease, Poland syndrome can occur sporadically or in families. Its treatment depends above all on the psychological repercussion of the disease.

## Highlights

• Poland syndrome is a rare malformative and congenital disease
• The etiopathogenesis of the disease is unknown hence the importance of any new research on the subject
• The diagnosis of the disease is not easy for inexperienced practitioners
• In the absence of associated malformation, the experience of the disease is relatively simple

## 1 Introduction

Poland syndrome is a rare malformative entity involving agenesis of the pectoralis major muscle and the mammary gland [1], [2], [3]. This condition has an estimated incidence (incidence however underestimated) of 1 birth per 30,000 births [2], [3]. The disease presents a predominance with respect to the male subject and in 1/3 of the cases affects the right side [1]. This malformation may or may not be associated with malformations of the ipsilateral thoracic limb [1], [2], [3]. Although, the first description was given to the French Lallemand in 1826, it was Doctor Alfred Poland, a British surgeon, who was the first to provide a complete description of this malformation [1], [2], [3]. In 1841, he described cutaneous-glandular, osteo-cartilaginous, and muscular thoracic damage [1], [2], [3]. In some more serious cases, an anomaly affecting the internal organs (lungs, kidneys and heart) has been reported [1]. The etiology of Poland syndrome remains unknown; the hypothesis of vascularization arrest during fetal development has been reported [1], [2], [3]. Familial cases have also been reported without, however, identifying a true genetic cause. The diagnosis and management of Poland syndrome remain controversial to date [2]. No consensus has yet been reached [2]. We report in our work a case of familial Poland syndrome of fortuitous discovery on the basis of the medical visit of a young Togolese man. This manuscript was written according to the rules of the SCARE [4].

## 2 Case report

**Fig. 1:**
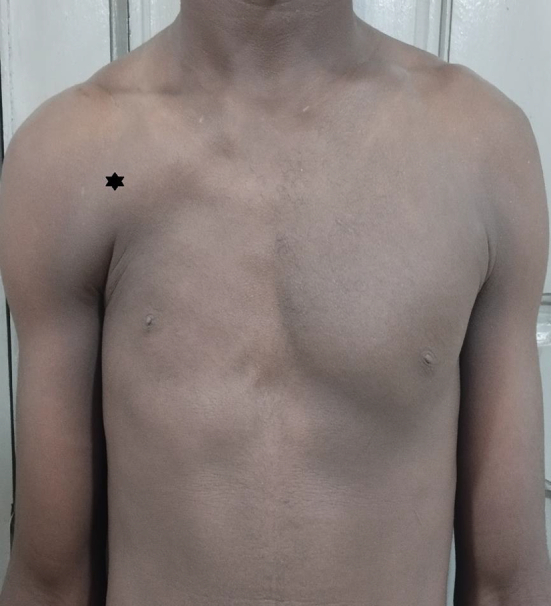
Patient with chest asymmetry with apparent hypertrophy of the clavicular bundle of the pectoralis major muscle ().

He was a 25-year-old student, with no known pathological history, received in consultation for a medical examination. Examination of the unclothed subject revealed a right thoracic depression responsible for thoracic asymmetry. Clinical exploration of the pectoral muscles revealed a right sub-clavicular depression (Fig. 1). This depression was related to agenesis of the sternocostal chiefs of the pectoralis major muscle with compensatory hypertrophy of the clavicular head of the said muscle. The skin at the level of the depression was thin and adhered to the chest wall. In addition, there was an absence of the right anterior axillary pillar. No abnormalities of the right thoracic limb were found. Ultrasound of the chest wall confirmed the absence of the mammary gland as well as the sterno-costal heads of the pectoralis major. The search for associated malformations (thoracic limbs, testicles, kidneys) was negative. The Doppler found symmetrical subclavian arteries of the same caliber. In the a posteriori interview, he has had this chest deformity since birth and the patient reports 3 other cases among his paternal uncles. He also plays sports regularly and does not report any limitations in his activities. The patient did not benefit from any therapeutic measure due to the absence of functional limitation, the absence of associated malformations and especially the absence of any psychological impairment. There were no functional limitations to his desire for a military career.

No proposal was made to the patient for the genetic diagnosis in relation to the high blow that this could entail. Similarly, we were unable to examine other family members concerning the condition. Their geographical distribution was a real obstacle for us with the costs of this (travel, accommodation and possible assessment). However, contact has been maintained with the patient in the hope of a future diagnostic program for rare genetic diseases.

## 3 Discussion

A rare congenital malformation, Poland syndrome associates several anomalies to varying degrees [1], [3], [5], [6], [7]. These abnormalities are cutaneous-glandular, osteo-cartilaginous, and muscular [1], [2], [3], [6]. They relate to the thorax and the ipsilateral thoracic limb [1], [5], [7]. This syndrome is named after Sir Alfred Poland, a British surgeon, who wrote its most complete description in 1841 [1], [2], [3], [7]. However, it was the French Lallemand who was the first to report a case of this syndrome in 1826 [3].

The etiopathogenesis of Poland syndrome has not yet been elucidated [1], [6], [9]. The disease occurs sporadically [1], [5], [8], [10]. Rare family cases of the condition have been reported, as was the case in our observation [1], [2], [7], [8], [10]. However, no real genetic evidence has yet been provided for this disease. Among the most popular theories, it would seem that the occurrence of Poland syndrome finds justification in the reduction of embryonic blood flow to the subclavian and vertebral arteries and their branches during the sixth week of amenorrhea [1], [2], [5], [6], [7], [11]. Thus, the degree and location of the alteration of this flow will define the severity of the syndrome [6]. Other theories have been developed, in particular the alteration of the mesoderm of the lateral plate after fertilization or even trauma, viral infections, maternal smoking during pregnancy [8].

The incidence of Poland syndrome is estimated at 1 case in 30,000 births [3], [11]. However, this incidence remains underestimated when we know that there are minor forms of the disease which remain underdiagnosed [3]. The distribution of cases is different depending on whether it is sporadic or familial form of the disease [3], [8], [11]. In familial cases, it is reported that the distribution of cases is almost uniform between the two sexes as well as on the laterality (right or left) [8]. On the other hand, in sporadic cases, male subjects are mainly affected [8], [9]. Similarly, in men, the right side is more affected, whereas in women, there is no predominance of one side over the other [8].

All patients with Poland syndrome have one thing in common: the absence of the sternocostal head of the pectoralis major muscle [3], [7], [8]. Thus, two forms of the disease emerge: a minor form relating to the isolated presence of thoracic anomalies and a major form associating thoracic anomalies with anomalies of the ipsilateral thoracic limb [3], [12]. The thoracic abnormalities involved in Poland syndrome are of three types: muscular, osteo-cartilaginous and cutaneous glandular [3], [12]. On the muscular level, agenesis of the sterno-costal heads of the pectoralis major muscle constitutes the most constant anomaly of the syndrome [3], [12]. This agenesis may be associated with hypoplasia of the other muscles of the thorax on the ipsilateral side [12]. Very often, agenesis of the pectoralis minor muscle is found [8]. Damage to the sterno-costal portions of the 2nd, 3rd, 4th, and 5th ribs constitute the main osteocartilaginous lesions [3], [7], [12]. It is a hypoplasia with extreme aplasia. Cutaneo-glandular abnormalities are made of thin with solid adhesions between the dermis and the chest wall [3], [12]. In women, there is breast hypoplasia or even aplasia responsible for breast asymmetry [7], [12]. Hair disorders may also exist [3], [12].

The diagnosis of Poland syndrome is exclusively clinical [2], [10]. The current consensus attributes agenesis or hypoplasia of the sternocostal heads of the pectoralis major muscle as a pathognomonic character of the disease [8], [10]. The clinical manifestation of this anomaly is infraclavicular depression and an absence of anterior axillary pillar [8]. The other associated thoracic signs may be breast (hypoplasia or agenesis), costal (depression related to costal agenesis, pectus carinatum or even excavatum), muscular (agenesis or hypoplasia of the pectoralis minor, serratus anterior, serratus anterior, dorsal) a thin skin more or less adherent to the thoracic wall [8], [10]. A major fact is that these signs are ipsilateral to pectoralis major muscle agenesis [10]. These thoracic signs may be associated with anomalies of the ipsilateral thoracic limb (asymmetry of axillary hairiness, radio-ulnar synostosis, syndactyly, brachymesophalangy) [6], [8], [10], [11]. In rare cases, Poland syndrome is found in a polymalformative complex. Cases of association of Poland syndrome with Mobius, Sprengel, Klippel-Feil, Adams-Olivier syndromes [1], [7], [8], [11]. Radiology does not play a very important role in the diagnosis of Poland syndrome [10]. If it can help confirm the clinical diagnosis, it is mainly in the search for associated malformations and in the therapeutic decision that it intervenes [2], [10]. The first-line exploration is chest ultrasound [2], [10]. It is accessible, inexpensive and non-irradiating [10]. CT and MRI are only used in the event of ultrasound abnormalities for further exploration [2], [10].

Poland syndrome itself does not involve any life-threatening or functional prognosis [3], [8], [10]. Most of the authors agree on this fact [3], [10]. Therapy draws its best support from the aesthetic damage and psychological damage that Poland syndrome can cause [1], [3], [8], [9], [10]. However, there is no other definitive treatment for the disease [3], [10]. There are only surgical and medical treatments for major abnormalities [3], [8], [10].

## 4 Conclusion

Poland syndrome is a rare congenital disease that can be sporadic or familial. If the embryonic origin is the most reported, the exact etiopathogenesis of the condition remains unknown. The distribution of cases according to sex and laterality varies depending on whether the form of the disease is minor or major. Its clinical diagnosis is relatively easy. The disease, in its typical form, is not functionally burdensome. His handicap is essentially aesthetic and represents an essential argument for his therapeutic approach. The treatment has several aspects including medical, surgical and psychological.

## Consent

Written informed consent was obtained from the patient for publication of this case report and accompanying images. A copy of the written consent is available for review by the Editor-in-Chief of this journal on request.

## Ethical approval

Ethical approval for this study was provided by the ethics committees of the Sylvanus Olympio University Hospital Center, Lomé, Togo on 18 January 2023.

## Funding

None.

## Author contribution

ETB: Study design and data acquisition

LAK, KET: literature review

ETB, LAK and DPRC: manuscript editing

All authors: manuscript review

## Guarantor

ESSOBIYOU Tamassi Bertrand.

## Research registration number

Not applicable.

## Provenance and peer review

Not commissioned, externally peer-reviewed.

## Declaration of competing interest

The authors declare that they have no known competing financial interests or personal relationships that could have appeared to influence the work reported in this paper.
